# Retrobulbar and Tongue Base Pyogranulomatous Myositis Resulting in Strabismus in a Dog: Case Report

**DOI:** 10.3389/fvets.2020.00360

**Published:** 2020-06-26

**Authors:** Alex B. Sigmund, Silke Hecht, Daniel A. Ward, Diane V. H. Hendrix

**Affiliations:** Department of Small Animal Clinical Sciences, University of Tennessee College of Veterinary Medicine, Knoxville, TN, United States

**Keywords:** granuloma, abscess, foreign body, eye, myositis, strabismus

## Abstract

A seven-year-old female spayed Australian Shepherd was presented for a 3-day history of left eye ventromedial strabismus, episcleral injection, protrusion of the third eyelid, miosis, and enophthalmia. Magnetic Resonance Imaging (MRI) identified lesions in the left medial pterygoid muscle and left tongue base. Cytology and histopathology revealed pyogranulomatous inflammation with rod-shaped bacteria and pyogranulomatous myositis, respectively. One month of oral antibiotics resolved both lesions. Repeat MRI showed a mild decrease in size of the left medial pterygoid muscle consistent with fibrosis. Clinically, residual, positional ventral strabismus remained upon dorsal neck extension, but all other ophthalmic abnormalities resolved. To the authors' knowledge, this is the first report of pyogranulomatous myositis causing this constellation of clinical signs and of repeat imaging depicting resolution of these lesions with therapy.

## Case Presentation

**Figure 1:**
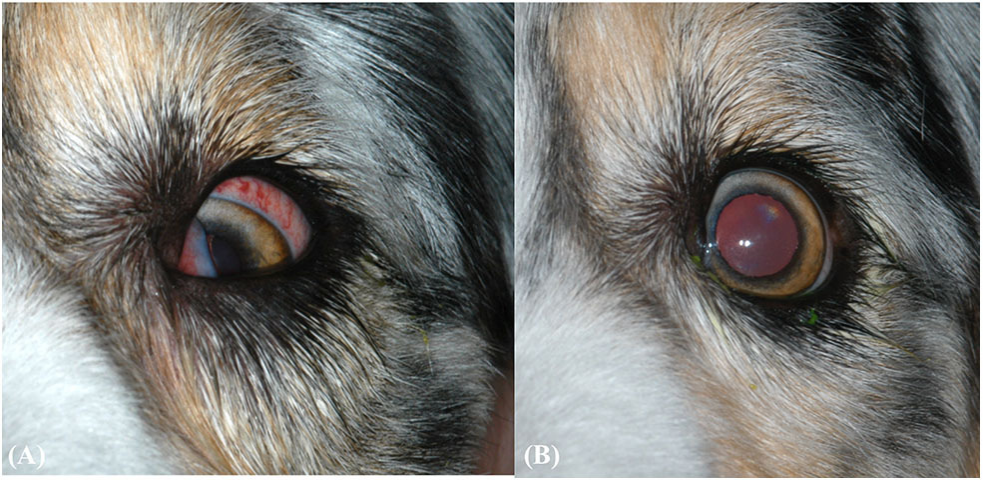
**(A)** Clinical ophthalmic findings at the second ophthalmic examination. Findings include protrusion of the third eyelid, ventromedial strabismus, and episcleral hyperemia. **(B)** Clinical ophthalmic findings after 1 month of oral amoxicillin/clavulanic acid for pyogranulomatous myositis. The eye has returned to normal positioning, but positional ventral strabismus remains during dorsal extension of the neck (not pictured).

A seven-year-old, female spayed Australian Shepherd was presented for severe chemosis and episcleral hyperemia of the left eye (OS). The remainder of the examination was unremarkable. Three days later, the eye had ventromedial strabismus with a positive passive forced duction test, and anterior movement of the third eyelid was restricted when grasped with forceps (Figure 1). The eye was visual and non-painful. The physical examination was normal. Two weeks later, the eye was enophthalmic and had mild conjunctival hyperemia and mild miosis. Under general anesthesia for imaging, the positive passive forced duction was marginally decreased.

## Image Acquisition

The dog was anesthetized and placed in dorsal recumbency for Magnetic Resonance Imaging (MRI) of the head using a 1.5 Tesla superconducting MRI system (MAGNETOM Espree™, Siemens Medical Solutions, Malvern, PA) equipped with a head and neck coil. Pre-contrast MRI sequences acquired included sagittal T2-weighted spin echo images (TR 4230 ms, TE 101 ms), dorsal short tau inversion recovery (STIR) images (TR 3570 ms, TE 41 ms, TI 160 ms), transverse T2-weighted images (TR 3530 ms, TE 101 ms), transverse T1-weighted images (TR 413 ms, TE 12 ms), transverse T2-weighted fluid attenuated inversion recovery (T2-FLAIR) images (TR 8420 ms, TE 75 ms, TI 2430 ms), transverse proton density (PD) weighted images (TR 2300 ms, TE 13 ms), transverse T2^*^-weighted gradient recalled echo (GRE) images (TR 1220 ms, TE 26 ms, flip angle 20°), and transverse T2-weighted TSE with Variable Flip Angle (“SPACE”) (TR 1100 ms, TE 123 ms). Post-contrast (Magnevist®, Bayer Healthcare Pharmaceuticals Inc., Wayne, NJ; 0.1 mmol/kg i.v.) sequences included transverse T1-weighted Volume Interpolated GRE images with fat saturation (“VIBE”) (TR 10 ms, TE 3.63 ms, flip angle 10°), sagittal T1-weighted images (TR 465 ms, TE 12 ms), transverse T1-weighted images (TR 413 ms, TE 12 ms), and dorsal T1-weighted images with fat saturation (TR 389 ms, TE 12 ms).

**Figure 2:**
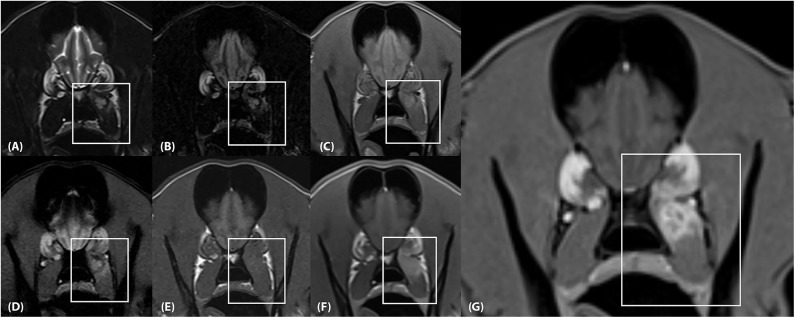
**(A)** Transverse T2-weighted, **(B)** T2-FLAIR, **(C)** PD-weighted, **(D)** T2*-weighted GRE, **(E)** T1-weighted pre contrast, **(F)** T1-weighted post contrast and **(G)** T1-weighted Volume Interpolated GRE images with fat saturation (“VIBE”) at the level of the frontal lobes. There is an ~2.5 × 1.3 × 1.2 cm (length × height × width) lesion located within the dorsal aspect of the left medial pterygoid muscle (box) which mostly follows the normal muscle contour aside from a focal region of muscle expansion in the dorsal aspect of the lesion. **(A,B)**, The lesion is mildly heterogeneously T2 and FLAIR hyperintense, **(C)** mildly heterogeneously PD hyperintense, **(D)** heterogeneously T2* hyperintense, and **(E)** T1 iso-to mildly hyperintense. **(F,G)** The lesion is strongly and heterogeneously contrast enhancing.

**Figure 3:**
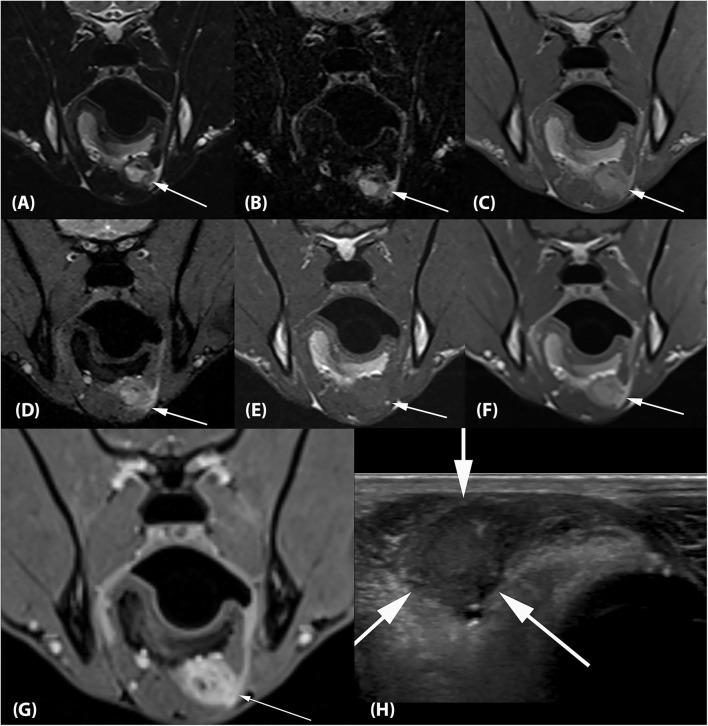
**(A)** Transverse T2-weighted, **(B)** T2-FLAIR, **(C)** PD-weighted, **(D)** T2*-weighted GRE, **(E)** T1-weighted pre contrast, **(F)** T1-weighted post contrast and **(G)** T1-weighted Volume Interpolated GRE images with fat saturation (“VIBE”) at the level of the mandibular angle. There is an ~2.0 × 1.3 × 1.8 cm (L × H × W) ovoid lesion associated with the left side of the tongue base with imaging characteristics identical to the lesion associated with the left medial pterygoid muscle (arrows). **(H)** Transverse ultrasonographic image of the lesion at the tongue base which is hypointense, fairly homogeneous, and well-circumscribed (arrows). The impression of the lesion being right- rather than left-sided is artifactual and related to a change in tongue position between the MRI and ultrasound examination.

Two separate lesions with remarkably similar imaging characteristics were associated with the soft tissues of the head. One was associated with the left medial pterygoid muscle immediately ventral to the orbital fissure (Figure 2). It measured ~2.5 × 1.3 × 1.2 cm (length × height × width; L × H × W) and mostly followed the normal muscle contour aside from a focal region of muscle expansion in the dorsal aspect of the lesion. The second lesion was associated with the left side of the tongue base, was ovoid in shape, and measured ~2.0 × 1.3 × 1.8 cm (L × H × W) (Figures 3A–G). Both lesions were mildly heterogeneously T2 and STIR hyperintense, T1 iso-to mildly hyperintense, PD hyperintense, did not suppress on FLAIR and did not exhibit susceptibility artifacts on T2^*^-weighted images. Pinpoint intralesional T1 and T2 hypointense foci were noted. The lesions were strongly contrast enhancing and mildly heterogeneous in intensity. The left mandibular and medial retropharyngeal lymph nodes were mildly enlarged and heterogeneously contrast enhancing. No intracranial abnormalities were identified. Ultrasound guided tissue sampling or surgical biopsy of the lesion at the tongue base was recommended.

Ultrasound examination of the ventral tongue was performed using a high frequency linear transducer (5–12 MHz, Epiq5, Philips Ultrasound, Bothell, WA). The tongue base lesion appeared well-circumscribed, hypoechoic, and fairly homogeneous (Figure 3H).

## Cytology and Histopathology

Cytology of a fine needle aspirate of the tongue base mass revealed pyogranulomatous inflammation and rod-shaped bacteria. An incisional biopsy of this lesion revealed pyogranulomatous myositis, possibly secondary to prior foreign body penetration. Foreign material was not identified within the submitted biopsy sample. A tissue culture was not done, but, in retrospect, should have been submitted. Sampling of the pterygoid muscle lesion could not be performed due to proximity to the maxillary artery.

## Treatment

The patient was placed on oral amoxicillin/clavulanic acid (15.6 mg/kg q12h, Clavamox™, Zoetis Services, LLC, Parsippany, NJ) for 1 month and the clinical signs resolved. Ten weeks after presentation, the ophthalmic examination was normal except for residual, positional ventral strabismus OS during dorsal extension of the neck (Figure 1).

## Follow Up

**Figure 4:**
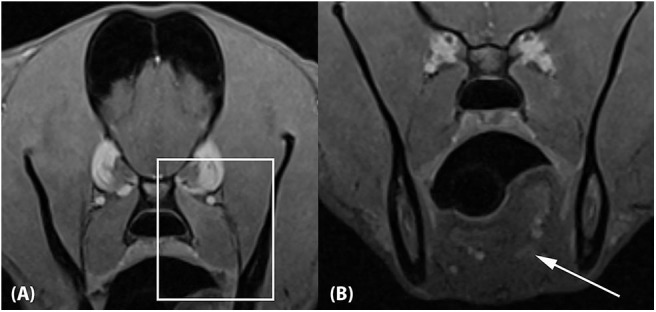
Approximately 10-week recheck MRI examination of the head. **(A)** Transverse T1-weighted Volume Interpolated GRE images with fat saturation (“VIBE”) at the level of the previous medial pterygoid muscle lesion and **(B)** the previous tongue base lesion. The lesions have mostly resolved, with mild residual contrast enhancement noted associated with the dorsal aspect of the left medial pterygoid muscle (**A**; box) and the tongue base (**B**; arrow). The left medial pterygoid muscle is mildly decreased in size compared to the right.

A repeat MRI examination was performed 10 weeks after presentation. The protocol was identical to the initial study. The previously seen soft tissue lesions associated with the left medial pterygoid muscle and the tongue base were largely resolved. Faint regions of altered signal intensity (minimal T1 and T2 hyperintensity and mild contrast enhancement) were still noted (Figure 4). The left medial pterygoid muscle was mildly smaller compared to the right. The mandibular and medial retropharyngeal lymph nodes were normal. No new abnormalities were identified.

## Discussion

This report describes a unique cause of ventral strabismus in a dog. Given the initial clinical signs of resting ventromedial strabismus, retrobulbar soft tissue pathology was suspected. Strabismus can be classified in many ways, such as resting vs. positional and restrictive vs. neuronal. A “resting” ventral strabismus is most indicative of a restrictive process, such as fibrosis or muscle impingement, that prevents proper relaxation of specific muscles. A “positional” strabismus is only seen in certain head orientations and most commonly occurs from a vestibular neuropathy (1). In the current report, ventromedial strabismus was present at rest indicating the reciprocal extraocular muscle was unable to move the eye against the restricted muscle. Passive forced duction testing confirmed a restrictive process as dorsolateral movement of the globe was not possible. There was also mild miosis indicating at least mild cranial nerve III (CN III) dysfunction. If complete CN III dysfunction alone was present, then miosis and ventrolateral strabismus would be expected. If there was just a neuronal process occurring, the globe would move easily on forced duction, and contracture of the reciprocal extraocular muscle would cause strabismus opposite the paretic muscle.

On presentation, the restricted globe movement, ventromedial strabismus, and miosis indicated a combination of a neuropathy of the branches of CN III and inflammation of the musculature. Once CN III exits the orbital fissure, several branches run through the orbit to innervate the parasympathetic iris and ciliary body muscles, the dorsal, medial, and ventral rectus muscles, and the ventral oblique muscle. The medial pterygoid muscle forms part of the posterior orbital floor (1). The intramuscular lesion in this tissue could have compromised branches of CN III and/or inflamed the ventral rectus muscle resulting in ventromedial strabismus and miosis. Since the remainder of the neuro-ophthalmic examination was normal and the clinical signs correlated with CN III dysfunction from possible ventral orbit inflammation, no other cranial nerve pathologies were suspected. Advanced imaging was therefore needed to investigate these tissues.

Two intramuscular lesions were appreciated on MRI. Differential diagnoses for intramuscular nodules and masses in humans include primary and metastatic neoplasia, hematomas, nodular myositis, sarcoidosis, crystal deposition and post injection changes (2). Dependent on the underlying cause, the MRI characteristics of the nodules and concurrent abnormalities are variable.

Reports of imaging findings in dogs with muscle lesions are limited. Inflammatory myopathies encompass a large group of heterogeneous disease processes of various etiologies including infectious and immune-mediated etiologies. Even though reported MRI features are variable, these conditions often cause multifocal patchy, ill-defined intramuscular lesions rather than distinct nodules as seen in our case (3–7). Abnormalities in canine myopathies may be unilateral or bilateral. With some inflammatory conditions (e.g., masticatory myositis and iliopsoas myopathy), changes may appear fairly symmetric (5, 8, 9).

Upon questioning, the dog's owners mentioned that the dog frequently chews on sticks, but they had not noticed any clinical signs commonly seen with acute penetrating oropharyngeal injuries such as retching, salivation, or pain. The pathology results in our patient suggested a prior penetrating injury; however, it is worth noting that foreign material was not identified on imaging or histopathology. It is conceivable that the ends of a sharp-edged stick penetrated both the dorsal and ventral lining of the oral cavity, introduced bacteria into the soft tissues, and caused the inflammatory lesions found on imaging without actually leaving foreign material behind. Alternatively, it is possible that foreign particles were left behind but were too small to be seen on imaging or histopathology. Little is known about the accuracy of MRI in detecting foreign bodies in soft tissues. In one experimental study, acute wooden foreign material appeared T1 and T2 hypointense to surrounding musculature (10). Even though CT and ultrasound performed better in the identification of foreign bodies in that study, the results have to be interpreted with caution as the study was performed on cadaver specimen, and foreign body induced soft tissue changes could not be assessed. Inflammatory soft tissue changes secondary to foreign bodies appear T2 hyperintense and are expected to highlight any foreign material embedded within the lesion (5, 7, 11). Other common findings with foreign bodies include fluid cavities and sinus tracts. The intralesional punctate T1 and T2 hypointense foci were most consistent with fibrosis or may have represented very small residual foreign body fragments not identified on histopathology.

Metastatic disease to muscle may occur secondary to sarcomas (e.g., hemangiosarcoma or fibrosarcoma), carcinomas (e.g., adenocarcinoma or squamous cell carcinoma), and round cell tumors (histiocytic sarcoma, lymphoma, or melanoma). Most reports of imaging findings with muscle metastases in dogs are based on whole body computed tomography studies (12, 13). On CT, muscle metastases appear as typically well-demarcated oval to round lesions with variable contrast enhancement. MRI findings in a dog with adenocarcinoma metastasis to the intertransversarius cervicis muscle included a uniform, T2 hyperintense and T1 isointense and uniformly contrast enhancing intramuscular nodule (14).

A repeat MRI was done to investigate if the resolution of clinical signs corresponded to resolution of the lesions noted on the initial MRI. The decrease in muscle volume and mild diffuse signal intensity changes seen associated with the left medial pterygoid muscle are consistent with fibrosis and correspond to abnormalities reported with other fibrotic myopathies in dogs (5, 7, 15). Fibrosis within this area is consistent with the clinical finding of persistent, positional ventromedial strabismus and a positive, passive forced duction test. The inability to dorsally rotate the globe most likely indicates a restrictive process. The initial passive forced duction test appeared to have improved 2 weeks later. This could have been due to partial consolidation of retrobulbar inflammation or due to the dog being anesthetized. Since the previously noted miosis resolved, there was likely consolidation of retrobulbar inflammation that decreased compression of CN III branches. Residual fibrosis of the medial pteryoid muscle and surrounding fascia likely resulted in the residual positional, ventral strabismus.

## Concluding Remarks

In conclusion, this report describes the MRI findings of a dog with pyogranulomatous myositis of the tongue base and left medial pterygoid muscle resulting in ventromedial strabismus OS. Rather than being ill-defined, these inflammatory lesions were distinct nodules. The current patient had a history of gnawing on various objects, including sticks. Therefore, both lesions could have occurred from an object penetrating the tongue base and retrobulbar space; however, no foreign material was found on histopathology and no traumatic event was observed. While no culture of the tongue base lesion was performed, cytology and histopathology revealed rod-shaped bacteria and myositis, respectively. Anaerobic and aerobic cultures should be performed for similar appearing lesions to help with diagnosis and guide antibiotic therapy. Given their appearance, both lesions were assumed to be from the same process. A one-month course of a broad-spectrum antibiotic resolved the lesions confirming this assumption. A repeat MRI examination showed absence of retrobulbar inflammation and fibrosis of the left medial pterygoid muscle correlating with resolution of previously noted neuro-ophthalmic abnormalities and only persistent, clinically evident positional ventral strabismus, respectively.

## Data Availability Statement

All datasets presented in this study are included in the article/supplementary material.

## Ethics Statement

Ethical review and approval was not required for the animal study because the report was written retrospectively on a patient treated with standards of care at a veterinary teaching hospital. Patient care, including diagnosis and treatment, did not include methods intended for research. Written informed consent for participation was not obtained from the owners because ethical approval and written consent from the owner were not needed for this report. Oral consent was given by the owner.

## Author Contributions

Conception and design done by AS, DH, and SH. Acquisition of data done by all authors. Drafting of the article as well as article revision done by all authors. Final approval of the completed article done by all authors.

## Conflict of Interest

The authors declare that the research was conducted in the absence of any commercial or financial relationships that could be construed as a potential conflict of interest.
